# The Social Basis of Vaccine Questioning and Refusal: A Qualitative Study Employing Bourdieu’s Concepts of ‘Capitals’ and ‘Habitus’

**DOI:** 10.3390/ijerph15051044

**Published:** 2018-05-22

**Authors:** Katie Attwell, Samantha B. Meyer, Paul R. Ward

**Affiliations:** 1 School of Social Science, University of Western Australia, Crawley, WA 6009, Australia; 2 Wesfarmers Centre of Vaccines and Infectious Diseases, Telethon Kids Institute, Subiaco, WA 6008, Australia; 3 Immunisation Alliance of Western Australia, Cockburn Integrated Health and Community Facility, Suite 14, 11 Wentworth Parade Success, WA 6164, Australia; 4 School of Public Health and Health Systems, University of Waterloo, Waterloo, ON N2L3G1, Canada; samantha.meyer@uwaterloo.ca; 5 College of Medicine and Public Health, Flinders University, Adelaide, SA 5001, Australia; paul.ward@flinders.edu.au

**Keywords:** vaccine acceptance, vaccine hesitancy, vaccine refusal, vaccine decision-making, social determinants of health, vaccine delay, vaccine confidence, habitus, social capital

## Abstract

This article is an in-depth analysis of the social nature of vaccine decision-making. It employs the sociological theory of Bourdieu and Ingram to consider how parents experience non-vaccination as a valued form of capital in specific communities, and how this can affect their decision-making. Drawing on research conducted in two Australian cities, our qualitative analysis of new interview data shows that parents experience disjuncture and tugs towards ‘appropriate’ forms of vaccination behavior in their social networks, as these link to broader behaviors around food, school choices and birth practices. We show how differences emerge between the two cities based on study designs, such that we are able to see some parents at the center of groups valorizing their decisions, whilst others feel marginalized within their communities for their decisions to vaccinate. We draw on the work of philosopher Mark Navin to consider how all parents join epistemic communities that reward compliance and conformity with the status quo and consider what this means for interventions that seek to influence the flow of pro-vaccine information through vaccine-critical social groups.

## 1. Introduction

After many years of research in the field, today’s scholars have a good understanding of the reasons parents reject vaccines and the aspects of vaccines that they fear [1,2,3]. We know that they distrust the expert systems which design and deliver vaccines [4] and that they may regard vaccines as an unwelcome and unnatural incursion into a ‘natural body’ which they view as unneeded or unbeneficial [5,6]. We also know that the vaccination behaviors of our social networks are a predictor of our own behaviors [7], so it is clear that our milieu matters to the decisions we make. What is missing is a theoretical account of how and why this is the case: how the beliefs of vaccine hesitant or rejecting parents are socially constructed, acquired and reinforced. This paper draws on Bourdieu’s notions of capital and habitus to elaborate these processes.

After introducing Bourdieu’s theory, the present paper explores vaccine questioning and rejection as social practices that hold value—identified as symbolic capital—in social networks containing parents who choose not to vaccinate, or who partially vaccinate their children. Using data from two Australian cities, we identify a parent’s choice regarding vaccination as a practice that they engage in as members of societies and communities for the purpose of inclusion or belonging. Our exploration suggests that those who design interventions—whether providers, public health officials or governments—need to understand these parents as socially situated. Parents’ connections to and investments in their peer groups may be a central reason that they value peer opinions over those of the experts with whom they disagree. We offer some suggestions as to how this might inform intervention design.

### 1.1. Bourdieu: Capitals Informing Our Choices and Identities

Questioning and rejection of vaccines by parents is just one of many contemporary practices illuminated by the application of the ideas of the sociologist Pierre Bourdieu [8,9]. Scholars have shed light on social practices from youth alcohol use [10], to moving schools [11], to sexual health [12] to preferences for different types of comedy [13], enhancing our understanding of how and why we behave in particular ways. Bourdieu’s key concepts and arguments regarding social learning and forms of capital can be summarized as follows.

Bourdieu argues that types of consumption, dispositions and social practices are stratified along ‘class’ lines, with some being ascribed greater moral and social worth (or to use Bourdieu’s lexicon, capital) than others [8]. Whilst Bourdieu originally referred to tastes, this has been extended to include things like ethical consumption and purchasing Fair Trade and organic produce [14,15,16,17]. Access to these patterns of consumption or practices are determined in part, by habitus. Habitus is an “embodied disposition shared by members of that class” [18] (p. 413) which is largely unconscious. For Bourdieu [8] (p. 32), each individual is born into particular cultural and class meaning systems; these systems code the body in ways of “standing, speaking and thereby of feeling and thinking”. Cumulative exposure to social conditions leads us to internalize these conditions, and hence we establish patterned responses to the external environment [19]. Practices such as purchasing organic food, or using forms of complementary and alternative medicine (CAM), have a practical logic, which Bourdieu identifies as a “‘feel for the game’ which works outside of conscious control” [20] (p. 528). These practices are, therefore, largely unreflective and socially located [10]. Vaccination practices (and related practices around CAM, ‘natural’ products etc.) are thus not only symbolic of, but are also deeply engrained bodily performances of, identity, class and social relations. These symbols and performances are transmitted, learned and reproduced in families, across generations and within groups and communities. 

Bourdieu identifies four types of capital [8], which are acquired both formally (e.g. education) and informally (e.g. family institutions) [11,18]:

1. economic capital
2. cultural capital (knowledge which is legitimised)
3. social capital (relationships with significant social others) and
4. symbolic capital (related to prestige).

Symbolic capital is “what every form of capital becomes when it obtains an explicit and practical recognition” [21] (p. 242). That is, capital becomes symbolic capital when “it is perceived as a positive sign” that signifies membership to an elite group and distinguishes one group from another [22] (p.57). Being the bearer of symbolic capital entitles an individual to “state with success what merits being known or recognised” [21] (p. 242). The bearer can speak for the group, and also impose values on others through creating the ‘official’ version of the world. 

We can thus understand vaccine questioning/rejection as socially situated, insofar as some groups—by virtue of access to economic and cultural capital—are more likely to be predisposed to adopt these vaccination practices [23]. Furthermore, vaccine questioning/rejection could be a form of symbolic capital within particular social groups. These practices are created and promoted by an internal elite within the social group, who have the power to define non-vaccination as desirable—a form of distinction. They may also possess the economic capital to fund the lifestyle associated with raising children ‘healthy’ enough to navigate the world unvaccinated [24,25,26]. Therefore, whilst we present data in this paper suggesting that vaccine rejection/hesitancy could be seen as a form of cultural and/or symbolic capital, we demonstrate that its central importance is as a differentiated but relational capital. It demonstrates who we are, and who we are not. 

We therefore explore the evidence that values-based and ethical consumption is viewed as a desirable and/or superior practice—a practice utilized to distinguish between groups on the basis of both vaccination practice and various other practices connected to ‘natural lifestyles’ [25]. Following Bourdieu, we argue that the ‘place’ of vaccine rejection needs to be understood as a reified or valorized practice within some social groups, but not necessarily others [27]. (In fact, in other groups—such as the medical profession or mainstream society—it is more likely to be questioned and dismissed as having low symbolic capital).

### 1.2. Vaccine Hesitancy and Rejection and Sociality

We believe that bringing this approach of Bourdieu to vaccine rejection is a novel contribution. Several scholars have already moved past or critiqued positivist and medical model thinking about vaccine hesitancy and vaccine refusal [28], recognizing the gendered and relational aspects of parents’ decision-making [6,26,28]. It is well-established that the notion of ‘informed choice’ has now, for many, become a social expectation to question evidence and advice around parenting, including vaccination. The cultural milieu in which contemporary parenting occurs places health as a moral responsibility and expectation of the individual. The rollback of the state has ‘responsibilised’ individuals—constructed as rational health consumers—to be accountable for their own-wellbeing [29]. However, such a construction belies not only the competing exhortations to ‘think for ourselves’ and ‘wisely follow expert advice,’ but the context-specific ways in which we receive and interpret such exhortations as members of social groups [30]. 

Streefland et al.’s [31] groundbreaking anthropological work introduced the notion of ‘local vaccination cultures’ which root acceptance and rejection in social milieu. Leach and Fairhead [32] explored the cultural grounding—both acceptance and rejection—in developed and developing world settings. Sobo [33] found that vaccine refusing parents at a Steiner (Waldorf) school in California took cues from each other and performed in ways that would be socially rewarded by their peers, including eschewing vaccination. Sobo’s research demonstrated how parents who enrolled their children at a Steiner school might become inducted to be vaccine refusers even if they had not questioned vaccines prior to enrolment. We suggest that this induction can be read as a desire to acquire cultural capital in a new social setting. Indeed, Sobo [34] later argues for interpreting vaccine rejection as a positively framed act of opting in (socially) rather than opting out (medically). ‘Fitting in’ is a factor in the decision to vaccinate or not, and contrary to former reporting of guilt or shame associated with being labelled anti-vax, vaccinating may be a basis for exclusion from specific social settings. Accordingly, in this article, we demonstrate broader social experiences, as well as perceived expectations of vaccine hesitant and refusing parents, exploring how being part of communities impacts vaccination knowledge, beliefs and behaviors. We argue that vaccine rejection (alongside and often due to other linked ‘natural’ parenting practices) may shape and/or change parents’ habitus, deriving from childhood or community settings. 

We show how vaccine rejection promotes stability and inclusion for parents in some contexts, generating a sense of belonging and having the ‘right’ capital in specific social settings or communities. It therefore takes the form of high symbolic capital within these social groups. Vaccine rejection can also promote difficulties in other contexts, leading to self-censorship and fears of judgement, blame and disclosure. In these social contexts, non-vaccination has low symbolic capital. Whether or not vaccine rejection has symbolic capital within a particular social group will be determined by its relationship to other forms of cultural capital within that community, which are recognized by members as linked and meaningful. Specific ‘alternative lifestyle’ behaviors and consumptive patterns are central here. 

Our research builds on the work of Attwell and Smith [35], which advanced the idea that that there is a ‘cultural style’ associated with questioning or refusing vaccines. Our work also extends Dan Kahan’s [36] postulation that—similar to identified ‘cultural cleavages’ (voting patterns, gun ownership, abortion)—specific communities would hold shared characteristics within which vaccine refusal would be socially and culturally meaningful, even though he found no discernible relationship between a negative orientation towards vaccines and political outlook within a large population sample. Attwell and Smith argued that there were already recognizable characteristics of vaccine hesitant and rejecting parents evident in existing studies: use of complementary and alternative medicine, specific beliefs and desires relating to low intervention births, a reification of nature and the natural, and a set of economic and social circumstances that enabled labor intensive practices, which parents saw as conferring protection on their children. They applied social identity theory to speculate that these recognizable characteristics enabled participants to identify each other as part of a valued in-group whose beliefs and social relationships became mutually reinforcing. They suggested that one might ‘discover’ oneself to be a vaccine refuser on the basis of sharing other lifestyle and values attributes with co-members of the group, even if one had not held such a view prior [35]. We present such a case in this article. We suggest that some parents attribute high symbolic capital to particular shared practices forming the basis of parental identity, and that they recognize vaccine questioning and rejection as one of these key practices. Thus, it is not simply that vaccine skepticism is a social norm that is hegemonic in some social settings, and parents follow the norm because they follow the(ir) crowd. (After all, we are exposed to a variety of social norms as we go about our lives, and someone with a vaccine-rejecting mother or best friend might reject their perspectives in favor of a doctor’s recommendation.) What we seek to explore here are the processes that encourage some parents to follow the lead of vaccine-critical peers. These processes are observable in particular lifestyle-based social groups, and they exert pressure that we can observe both when individuals ultimately choose to reject vaccines, and when they accept them despite the social cost. 

## 2. Materials and Methods 

We analyzed data from two Australian cities: Fremantle, Western Australia (WA) and Adelaide, South Australia (SA), where we interviewed parents as part of two separate research projects. Both studies sought to understand parental vaccine hesitancy using a qualitative methodology with semi-structured interviews. The Fremantle study had an additional agenda, to be elaborated subsequently, which necessitated recruiting another cohort of parents who identified as living an alternative lifestyle, had vaccinated their children, but did not feel comfortable speaking about it socially. In bringing the Fremantle and Adelaide studies together, we provide common ground in methodology and purpose, but also some useful differences in terms of parents’ outlooks and experiences, as we elaborate below.

Adelaide interviews were conducted between October and December 2015 with parents in areas identified as having low immunization coverage rates [37]. Parents were recruited at a suburban organic community market and by snowballing and were screened to ensure that they met the study inclusion criteria of refusing or delaying recommended vaccines. Fremantle parents were interviewed between September 2013 and April 2014 from postcodes surrounding the City of Fremantle, which also had low immunization rates [37]. They were recruited through posters, advertisements in the local newspapers, social media and snowballing. Participants were also screened prior to interviews to ensure that they met the study inclusion criteria of identifying as living an alternative lifestyle, refusing or delaying recommended vaccines and having a child under five, but not being anti-vaccination, or fully vaccinating but remaining quiet about this in social interactions. Flinders University Social and Behavioural Research Ethics Committee and The University of Western Australia provided ethical approvals for the projects, under project number 6976 and permit RA 4/1/5890 respectively.

In total, 32 parents were interviewed, 12 from Fremantle (interviewed by KA) and 20 from Adelaide (interviewed by PR; see acknowledgments). The majority of participants were women (*n* = 28). The age range of parents was 24–50 years, with 19 parents aged between 36 and 42. Two thirds of the parents held a university qualification. The participants included 12 parents who had never vaccinated their child(ren), 5 who had commenced vaccinating but ceased, 7 who were currently delaying or partially vaccinating, and 5 parents who had previously delayed but who were now up-to-date. Three Fremantle parents in the study had vaccinated according to the schedule and were recruited to share their experiences of being vaccinators in an alternative community; a former delayer and—to an extent—some selective vaccinators also filled this function. 

Interviews were transcribed verbatim and coded and analyzed in NVivo 10 (QSR International). For the purpose of understanding the social practices shaping vaccination decision-making, we sought to determine how parents’ individual interests relate to the interests of the social group, and to the strategies they reported using to accumulate capital employed by people within the group (i.e. how these interests reflect or become sources of capital). To tap the parents’ social experiences and sense of identity, we triangulated the parents’ explicit representations of how their vaccine decisions linked to other lifestyle practices; the framing of alternative and social practices synthesized by Attwell and Smith [35]—themselves drawn from earlier influential studies, notably Rogers and Pilgrim [38] and Reich [26,39]; and our own lived experiences in such communities [40]. We therefore coded for explicit links to alternative lifestyles and philosophies, looking for noteworthy themes such use of labels like ‘alternative’ (and explication of what this meant), and practices like CAM use, breastfeeding, organic food and childbirth. While we had analyzed some of these data in previous papers as constituting parents’ ‘toolboxes’ for raising unvaccinated children, here our approach was to look at how they constituted communities of practice. We also coded for the influence of family of origin and the influence of friends and social networks, the latter as they pertained both to general views on health and lifestyle and specifically to vaccine decision-making. Separately, we coded for parents describing the power and impact of their social identities and networks more broadly in their vaccination and lifestyle decisions. We had such vast amounts of data on this that we explored the specific formation and interplay of ‘us’ and ‘Other’ (insider/outsider) identities in a separate publication [27]. Our key purpose in this current publication is instead to analyze, using the parents’ experiences, their social practices pertaining and linking to vaccination informed belonging and exclusion through the generation or destruction of symbolic capital in established networks and communities.

While both projects in this merged dataset probed for parents’ social experiences as they pertained to vaccination, data from Fremantle was particularly important because of the context of the research. In addition to exploring the position of vaccine hesitant parents as a research project in its own right, the Fremantle study pre-tested and then evaluated a pro-vaccination campaign run by a community group to specifically target parents who lived an alternative lifestyle. The “I Immunise” campaign, conducted by the Immunisation Alliance of WA, was a social identity based behavior change intervention using vaccinating role models carefully selected from the Fremantle social milieu. Role models were selected on the basis of practicing recognizable behaviors that related to the social grouping: baby-wearing, home birthing, whole-food eating and cloth nappy (diaper) usage. The aim of the campaign was not only to encourage hesitant parents to feel more confident in vaccinating, but also to change community conversations by encouraging vaccinating parents in alternative communities to speak up about their vaccination status and the reasons for their decisions (for more on this, see [40]). To this end, it was necessary to recruit into the study some parents who did vaccinate but felt uncomfortable speaking about it in their social networks. As noted, three such parents were recruited. In order to test the campaign concept and refine its delivery and messaging, Fremantle parents were interviewed before it ran. Some of these parents, as well as new participants, participated in the evaluation afterwards, describing its impact on their social world. Consequently, Fremantle interviews probed for the construction of identity, lifestyle, peer relationships and experiences in detail, whereas these phenomena emerged in the Adelaide data. Some Fremantle participants discussed their perceptions of the campaign as it related to their place in the community, and the impact of the campaign on their social networks after it ran. Many responses were relevant to showing how vaccine attitudes propagate through lifestyle and identity groups. 

In examining the role of the social in parents’ development of vaccine hesitancy or their rejection of vaccines, it is important to describe how we define and understand parents’ social experiences as they pertain to vaccination. We have focused on experiences in which participants encountered negative views towards vaccination in their milieu, rather than (for example) going looking for vaccination information online. The latter could also be regarded as a social experience in that one is engaging with material written and prepared by others, but we are interested in experiences that occur through face-to-face communal interactions.

Both Fremantle and Adelaide parents’ vaccine trajectories were mapped as part of the analysis—some parents had originally vaccinated but ceased, some had never vaccinated, some were now up to date with vaccination, and some had been highly hesitant but had always vaccinated. These differences were identified at the onset of analysis as important to the parents’ location within social networks, and their experiences as influencers and recipients of social pressure. Regular discussions within the research team guided the coding and analysis process at all stages.

## 3. Results and Discussion

Participants, particularly in Adelaide, deeply invested in alternative lifestyles, which they saw as supporting the health and immunity of their children. The parents’ natural births, long-term breastfeeding, gentle parenting, organic food consumption, eschewing of biomedicine and embrace of CAM were key rationales provided for their decision to reject some or all vaccines [25]. We have already reported how their trust in CAM and natural remedies (accompanied by a distrust of Western medicine including vaccines) had a social dimension, acquired through childhood immersion in many cases, or through trusted friends, family members and employers [41]. Although vaccination was constructed by participants as an individual decision, our data speak more widely to how parents were often socialized throughout childhood and further immersed during adulthood in alternative lifestyles. Their practices and dispositions formed through a continual exposure to and acceptance of a ‘natural’ lifestyle—this almost unconscious state of being that Bourdieu terms ‘habitus’ [9]. Hence for some participants, childhood immersion in an alternative lifestyle was a marker for their present-day habitus, what social grouping they belonged to (their social capital), their rationalized reasons for non-vaccination (cultural capital), and what ‘people like them’ would do with regard to vaccination and other highly valued parenting practices (symbolic capital).

Analysis of our data explains the various forms of capital used by participants in both validating their parenting practices and distinguishing themselves from others (with different parenting practices). We also show how parents, during their vaccine journey, have moved within and between different social groups and how this has affected their sense of identity and habitus. Here we draw of the work of Ingram, who provided a nuanced analysis of habitus, arguing that it is not a static ‘iron cage’, but rather that in certain circumstances is pulled, pushed, reshaped, sometimes causing problems for people. Ingram provided a detailed analysis of working class boys who became educated in elitist Grammar schools in Ireland [11], suggesting that ‘habitus’ can change when people move (or are forced) between social groups. She found processes of ‘destabilized habitus’ (no one really knowing who you are), ‘habitus tug’ (people being pulled by the expectations/rationalities of people in different social groups) and ‘disjunctive habitus’ (when the divided habitus causes problems). In analyzing our data, we recognized the analytic validity of Ingram’s taxonomy. However, we also recognized that some of our participants did not necessarily end up with a disjunctive habitus, but rather found themselves in a social group where they felt supported, validated and ‘like-minded’—which we designate a new category called ‘accepted habitus’.

### 3.1. Destabilized Habitus

Almost all parents had experienced a shift in parenting practices, dispositions and worldviews, often as a result of pregnancy and childbirth, but sometimes due to an illness of their child or perceived reaction to vaccinations. This shift started with a questioning of their current parenting practices (which were similar to other parents in their social group at the time) and a quest for new practices, which were more similar to those in a different social group. This process may be understood in terms of a destabilization of habitus—an initial questioning of why they did the things they did, followed by a search for new and improved ways of doing things. We consider this to be a ‘habitus tug’ from a different social group—one which included practices internally afforded high symbolic capital, such as more natural, ethical, environmentally friendly and less ‘clinical’ practices. 

For some participants, pregnancy and birth experiences were a pivotal point that made them question their previous/current practices and consider new/alternative ones. These experiences moved them from a ‘mainstream’ to an ‘alternative/natural’ social group, which included vaccine questioning. This new social group provided the social and cultural capital for some parents to feel part of what Beegan and colleagues call an “imagined community of like-minded others” leading to a sense of being part of a moral collective [42] (p. 766). Some participants did not necessarily have the ‘natural’ or ‘alternative’ habitus—they described themselves as mainstream prior to pregnancy—but various experiences jostled them and made them consider alternative pathways. 

For some parents, questioning or rejecting vaccines was itself the marker of high symbolic capital which facilitated them becoming part of the ‘alternative’ social group—a marker of distinction. Kavita, after initially complying with biomedicine via her private obstetrician, “started aligning [herself] with other mums who were saying, ‘Oh yeah, my baby gets sick after every vaccination too’ and then people saying ‘Actually my baby’s had some fairly severe reactions to immunization.’” 

Kavita’s destabilized habitus and habitus tug from and entry into a “resistant epistemic community” [30] coincided with her son being an undiagnosed celiac, and her development of distrust when her child health nurse suggested to a group of mothers that infant formula might help their babies sleep better—a practice Kavita afforded low symbolic capital. Kavita ultimately went on to become an important actor within her (new) social group:

“I think it’s sort of a journey that you start on, and you think, ‘Okay, what do I want my child to eat? Yes, I want him to eat organically.’ You do a bit of research, and you say, ‘Yeah, it does seem that it is a better way to go and, hey, the food tastes better as well.’ And you sort of start an organic market because you think that seems like a nice hobby to get involved in. Then it becomes a fulltime job and you have to call it business after three and a half years…” 

On this basis, we suggest that parents may come to know themselves as members of social groups through practices afforded high cultural capital that they share with other members, and ‘discover’ vaccine rejection as a form of high symbolic capital this way. However, attitudes towards and experiences with vaccination may also themselves be the lifestyle attributes that introduce parents to their social grouping. In this way, the ‘vaccination decision’ can be either the start or end point in the journey, but is nevertheless a marker of distinction between groups. In Kavita’s case, organic food was a clear reason for her destabilized habitus, and she became part of a social group including parents who shared her growing suspicions regarding the impact vaccines were having on her child. On her movement between social groups, she then came to adopt other markers of identity and behavior—a new habitus and ‘doxa’—Bourdieu’s notion of an unconscious, unquestioning common sense. 

The story of Anna, below, reveals that one may not ‘know’ oneself as a member of a social group until connected by specific and recognizable practices afforded high cultural capital. For Anna, this was a commitment to a natural lifestyle as a form of symbolic capital, which she then discovered (i.e. she was previously unaware) that she shared with vaccine refusers. This rather unconscious and non-planned alignment with vaccine rejection connects to the principle of habitus in Bourdieu’s work, and Anna’s story outlines the relatively unreflexive process of destabilized habitus to habitus tug. 

“…I must have come across something that made me aware that there is a choice [to reject vaccines] and that some people do make the choice, and that intrigued me. Why would they do that? Then I was noticing more and more that those that did not vaccinate were more along the lines of, you know, a more natural approach.”

Since Anna identified herself with the social group (natural approach) of vaccine rejecters, this was the catalyst for her beginning to read and investigate, a path that would ultimately lead to vaccine refusal. She observed what people ‘like her’ were doing and followed their lead—she regarded these practices as having high symbolic capital. This encapsulates the ‘habitus tug’ [11], a move from not knowing who she was—a destabilized habitus or a kind of liminal state—to a move away from her previous social group, once she recognized her new forms of symbolic capital. She identified with high symbolic capital (natural parenting styles) within a different social group to her own (destabilization), which led her to search for and undertake practices with high cultural capital within the new social group, enhancing the bonding via social capital within the new social group. 

Meanwhile, for Sonya, a vaccine delayer who had her two sons in Santa Cruz, California, her social group was a geographical given. 

“Santa Cruz is very much a hippie area. You’re either a redneck, a feminist, a lesbian or a high-tech person. So very much I was indoctrinated into the hippie ways when my kids were born, so going to parent groups and whatever. There were lots of people wearing flowing Indian gowns and... I did prenatal yoga and stuff. I was exposed to all that stuff right from the very beginning and there’s no other way to be in Santa Cruz because that’s just how Santa Cruz is; it’s that kind of place.”

At another point in the interview, Sonya declared, “I tell you what, if you tried to bottle feed in public in Santa Cruz you would get the filthiest looks on the planet”, elucidating the problematic nature certain practices accrue within ‘different’ social groups—blame, stigma and what Bourdieu calls ‘symbolic violence’. Sonya described this as part of the background as to why she had delayed vaccinations for her two sons. For her, the relationship between the social identity of those around her and her own vaccination practices was self-evident—‘the rules of the game’. She had done what people like her in her community did, pursuing practices with high cultural capital to accrue more social capital in a stable social group.

### 3.2. Habitus Tug

Another example of ‘habitus tug’ was described by Pippa in Adelaide, who started vaccinating according to the schedule but then became a selective vaccinator. Pippa attributed this to a shift in her approach to the birthing model between her two children. 

“[I]n the intervening time, between [first child]’s birth and my second pregnancy, I had begun to question a lot more the medical model about how we approach pregnancy and birth. That sort of flowed out fairly naturally then into other mainstream medical decision making. …[B]y the time I was pregnant for the second time … I chose to have a home birth, and that led me to sort of question a lot of the standard antenatal care. That led me to question a lot of aspects of standard medical ways of handling birth, obviously choosing to home birth, and then also I had a lot of questioning then about our approach to health care, I suppose, for children, after...”

The questioning by Pippa reveals the marker between the two social groups—leaving one and entering another [11]. As Pippa continued, the social aspect of this questioning became clearer:

“…I joined up with a couple of consumer groups around maternity care and I met midwives, I met women who’d home birthed and that really helped—and I read a lot of research papers about the safety of home birth so that helped me to feel that I could make an informed decision. …Just because I’m pregnant, it doesn’t have to mean that I have the glucose tolerance test, you know, there are other ways I can sort of measure my—what’s happening with me in the pregnancy. I don’t necessarily have to have the GBS [Group B streptococcus] swab, there are other things that we can do, or I can sort of weigh up the risks. So that’s what led me then when I had [younger child] to think maybe I want to reconsider the vaccine stuff.”

A shift from mainstream to alternative via birth practices can also be traced in Betina, who fully vaccinated her first daughter, born in the private obstetric system. Betina went on to delay vaccines for her second daughter, who was born in a group midwifery practice setting. Betina described how, in what we identify as a new social group, there was far more openness about critiquing and questioning medical interventions like the administration of antibiotics to birthing mothers who had previously tested positive for Strep B. These mothers’ critique or rejection of standard biomedical care (starting from decisions to homebirth or birth in midwife-led settings) demonstrates the relationship between low-intervention birth choices and vaccine hesitancy or rejection found by other scholars [43], a relationship that also extends to rejecting other biomedical interventions such as the Vitamin K injection [44].

While in these cases the mothers presented a trajectory, or habitus tug, in their thinking as they became socialized into different relationships to biomedicine and birth, Ariana, a vaccinating Fremantle doula, suggested that a questioning, critical approach to life would—by default—inform birthing decisions. “If you’re an alternative person, and you want to know what your best choices are, going to the mainstream pregnancy and birth system are not the best outcome, in my opinion.” She believed that was a factor in her own questioning of vaccines, and her framing is noteworthy. It suggests that knowing oneself via habitus to be an ‘alternative person’ means knowing how alternative people behave (akin to doxa), with questioning the medical establishment’s approach to birth part of that behavior, and questioning vaccines the logical next step. The practices around questioning/science and making ‘alternative’ choices to the mainstream articulate forms of cultural capital that are held in high esteem within this social group. These practices highlight that a person has ‘done their research’ and made what, to them, is the appropriate choice around vaccination. Questioning vaccination thus has high symbolic capital in this social group, and becomes a marker of distinction from vaccinating parents.

### 3.3. Disjunctive Habitus

Not all parents who found themselves participating in social groups via other high capital practices expected or were comfortable with the idea of rejecting vaccines, as Fremantle participant Marianne revealed. Her ‘habitus tug’ was home birth, but she found vaccine rejection to be a jarring fit within the social network she entered—a form of ‘disjunctive habitus’.

“It surprised me, when my husband and I decided to have our child at home and started exploring attitudes around parenting and pregnancy and health, and that sort of stuff … that people were anti-vaccination…. That really came out of left field. I guess I had perceived [that] it was something that was more associated with, perhaps, poorly educated people, rather than the highly educated people that were certainly engaged and around us with the home birthing practices.”

Marianne found herself with a foot in two social groups, but not fully comfortable in either. Her home-birthing experience left her slightly at odds with the ‘mainstream’ social group, but she was not prepared to adhere to the non-vaccination practices in the alternative social group. In many ways, Marianne displays a kind of ‘between and betwixt’ position of not really fitting in (destabilized habitus), but it also caused problems for her (disjunctive habitus) [11]. This fits with Bourdieu’s construction of social fields, which involve class-based tensions between social groups who co-exist in geographical or social spaces. Although our ‘natural’ and ‘alternative’ social groups do not fit the classical taxonomy of ‘class’, they certainly exhibit patterns of distinction which define them/us and have the power (symbolic violence) to impact negatively on the bearer of low symbolic capital (or positively for high symbolic capital).

Fremantle participant Casey, raised unvaccinated and hence having a habitus primed to be hesitant about vaccines, described a short delay in her child’s twelve-month vaccines not for reasons of fear, but because her mother was assisting with childcare so that Casey could work part-time. “I put it off for, like, a couple months, and made sure he was well settled before I did that one. He was staying with my mum. And I didn’t want him to get really sick with my mum, who would, like, judge me.”

This quote clearly reveals the difficulties associated with making vaccinating decisions in social fields that are not homogenous around non-vaccination, an example of ‘disjunctive habitus’ [11]. Casey’s mum was part of a social group in which vaccination has low symbolic capital, thereby influencing Casey to delay vaccinating through fear of judgement, or as Bourdieu called it, ‘distinction’ between one group and another. 

Similarly, accounts of some of our other participants demonstrate an inherently social and socialized journey to vaccine decision-making, often culminating in refusal. Movement from one social group into another (for whatever reason) necessarily involves questioning past, current and future practices, potentially leading to ‘habitus tug’ if new practices and dispositions are adopted and old ones eschewed. For Charlotte in Adelaide, her induction into vaccine questioning came in pregnancy through her exposure to a group called Future Families in Adelaide.

“…It was a not for profit organization run by volunteers and … it was a bit like a market for finding out different things about alternative ways to bring up children…. VISA [Vaccination Information Serving Australia—an anti-vaccine organization] were there… There was information about modern cloth nappies, information about different types of foods and all that sort of stuff. That’s ultimately also where I found out about Steiner education …. So we found out about the other side to vaccination there, and the more I heard, the more I kind of went, ‘Well, I don’t really know if I want to vaccinate my children’.”

Steiner education—the method that Charlotte chose after her exposure to Future Families, was also the school of choice for non-vaccinating Fremantle participant Angela’s children. Angela continued to question whether she had made the right decision with regard to vaccination. In terms of how she navigated these uncertainties socially, she characterized her experience as such:

“We’ve been doing Steiner, and then homeschooling and [alternative regional community], and then Steiner again. They are alternative communities. So I don’t speak up [about my doubts over not vaccinating] because there is almost no function to it. If you don’t immunize your child in this sort of thing, you’re a good parent. They’re [the people in in those communities] probably clearer than I am about why it’s better not to immunize your child.” 

Angela exhibits a disjunctive habitus, whereby she sits between two social groups within the social field, although feels unable to speak up against the ‘alternative’ social group due to its power to make her feel like a bad parent. She still questions her vaccination decisions, linking to a different social group where vaccination holds high symbolic capital, but also engages with the social group in which non-vaccination holds high symbolic capital. Her involvement in both groups within the social field creates difficulties for Angela, due to their almost polar opposite definitions of practices imbued with high symbolic capital. 

The decision to proceed with vaccination was no easier for Fremantle parent Jennifer, who described a high level of anxiety and continued self-doubt, which was a product of a disjunctive habitus. “A lot of my friends that I spend time with don’t immunize their kids, and I just worry that maybe I am doing the wrong thing (by immunizing them).” Jennifer also struggled with social engagement on the issue. 

“This is one topic where I feel it’s really hard to get a grasp on what the facts really are, and so I tend to avoid arguments with people because quite a few of my friends are really strongly anti-vaccine. And they will say their opinions to me, and I find it really hard to defend myself. Because what they are saying makes sense when they are saying it, and I’m like, ‘Well, I think you’re wrong,’ but I can’t prove that they are wrong. So it definitely causes conflict, but it hasn’t cost me any friendships. We just agree to disagree.” 

Tabitha, who followed a delayed schedule for her children, reflected on her own lack of desire to speak up about vaccinating within one of the social groups she engaged with, as a result of the difficulty in ‘going against’ the practices with high symbolic capital such as non-vaccination. She suggested that a “really strange outcome” of the “whooping cough epidemic in Fremantle” was that she felt more comfortable sharing her status as a (partial) vaccinator. “I live in the crux of where the situation is happening. The actual disease itself makes me more confident to say that in an alternative community.” 

Fremantle selective vaccinator Amanda seconded the sentiment that it was not easy to be a vaccinator in the Fremantle alternative parenting community. “It is definitely uncomfortable in a lot of the community situations to say that you have vaccinated.” 

Confident vaccinator Marianne concurred, explaining that she had experienced a lot of heated debate and discussion and almost ostracism about, “Why would you do that? Why would you feed your child toxins?” and, “They won’t get sick, those diseases are all but eradicated.” She stated: “I guess I didn’t talk about it very much. I was silent about what we did. I just went and did it. And I guess that felt… not dirty or wrong… but almost a bit of cloak and dagger, rather than going. This is just a normal thing we do, is immunize our kids.”

Fremantle parent Amanda spoke most poignantly about social influences and social pressures when it came to vaccination. Amanda was a young mother who had found herself pregnant, made redundant and living in a new town in a short space of time. Amanda had accepted all vaccines for her son except varicella, feeling that she would prefer him to acquire the immunity for that disease via infection. She had come to question vaccines—and to reject this particular one—after seeing friends post articles on social media. “I never really had concerns before that… [I]t’s more about the people in my community presenting information than I sought it out.” This represents a kind of passive destabilized habitus, moving into a habitus tug. 

Amanda reflected upon the people in her social field who were rejecting vaccines and how this provided an accepting environment for discussion about the various reasons behind their decisions. 

“I think most of the people are pretty intelligent, pretty educated, internally motivated people. … I suppose, being alternative, you have said that you’re making your own path, that you are not following the people. And while I do think that perhaps there is some sort of alternative conspicuous consumption—that there might be a little bit of skewing of direction—… I still feel [alternative] people are quite independent, and more so than the general population … And … they all have their own very individual reasons for not vaccinating. Like, there was one particular issue that got them. Like, you get a gazillion reasons on Internet why you shouldn’t, but there has never been, like, the whole room doesn’t immunize for the same reason. This is why it’s so hard to talk about. You can get into these arguments about “that is not a real reason to vaccinate,” and they’re saying, “Yeah, I’m open my kids to catching the disease, and that is how it should be,” “because it’s a myth and it’s just hygiene that got rid of these diseases,” it’s an absolute minefield to talk about…”

The previous quote suggests the difficulties incurred in raising questions about vaccine acceptance when non-vaccination is imbued with high symbolic capital by one social group. When Amanda began to use her Facebook page to share and engage with the “I Immunise” campaign in Western Australia, which was ultimately trying to improve childhood vaccination rates, she revealed that she “lost friends”. 

“… I brought up the moral issue of vaccinating and protecting the vulnerable members of the community, and people really didn’t like that. And that seemed to be a flashpoint for them, a cognitive dissonance…. I was [troubled by it] at the time, but now I feel like I’ve made different friends that are a bit more… critical thinking, and who understand that a bit more, but still share my parenting philosophies, mostly. …” 

These findings echo those of the parents in Sobo’s Steiner school study who went against the local social norm and vaccinated. In that study, parents reported not speaking up about vaccinating because they did not want to reveal themselves as not following the predominant culture and ethos of the school. Sobo comments, “paradoxically, keeping mainstream behaviors secret supported belief in their rarity” [33] (p. 12). This supports the ‘power’ of practices held with high symbolic capital, and how symbolic violence may be used to castigate and censor parents who, for whatever reason, do not conform to such practices.

### 3.4. Habitus Acceptance

The previous section highlighted the movement between social groups, and some of the difficulties incurred by parents during this move. However, for our participants in Adelaide, the social experiences were far more positive. Here, the data collection began at an organic market, which we can recognize as a key social group informing the parents’ identities and relationships. Research on farmers’ markets argues that they are constituted by “local, moral, ethical and environmental discourses,” with attendance connoting the consumer as a member of a network with shared moral values around “locality, artisan production techniques … and socio-environmental sustainability” [45] (p. 417). As such, farmers’ markets are premised on a “shared collective identity and lifestyle” [45] (p. 423), which was certainly the case for the Adelaide participants. 

For those who had made the decision not to vaccinate, then, their social groups proved to be stalwart supports. These social groups provided information regarding which health professionals would sign Conscientious Objector forms so that the parents could still access government benefits despite not vaccinating. They also allowed parents to rest assured that their children’s non-vaccinated status would not be an issue at their Montessori or Steiner schools. Natalie presented the large number of vaccine refusers at her child’s school as following a ‘common sense’ form of child health promotion:

“He’s at Montessori, which is not as left field as Steiner, but not as right field as normal, I guess. Yeah, so that was all fine, you know. If everyone’s got the exemption form, no-one bats an eye… It’s a really good school as far as healthy eating and that sort of thing, so I know the parents are aware of common sense things to keep their kids healthy”.

Supportive social groups also meant that one could be basically secure in knowing that one’s beliefs were accepted, as outlined by Kavita, “I guess I’m surrounded by a cohort of people now who are very similar, so I guess we support each other in that way”. This support was important, because, as Charlotte noted: “That’s not to say it has been easy, because you come up against a lot of people that do vaccinate. I mean in this community a lot of people don’t, but there’s still people that do.”

Once the parents came together in their social group of natural minded or questioning parents, critical of biomedicine and other ‘mainstream’ conventions, they were able to describe in meaningful and lucid ways the power of these social networks in their identification and decision-making with regard to vaccination, and also their ability to talk about vaccination within their social networks. The kind of lifestyle packaging of various forms of cultural capital that Charlotte experienced through Future Families was offered as an explanation by other participants as to how they saw themselves becoming part of a social collective. “Likeminded people will always come together,” professed non-vaccinating father Evan in Adelaide, another way of referring to bonding social capital, whereby habitus is not questioned or tugged, but accepted and valorized. Kavita described with acuity the high cultural capital practices she observed at the organic market:

“When I think about the organic market—and I’ve said it time and time again—they are baby wearing, breastfeeding, home schooling, non-vaxing ... mummas who eat organic food … It generally is the mums … They are the primary carer, they are the ones making the decisions most of the time. They are aligning themselves with that similar philosophy of, ‘Let’s grow our own food. Let’s breastfeed baby until they want to be breastfed. Let’s introduce a different type of food, not necessarily rice cereal and other grains. Let’s introduce other types of food. Let’s not introduce sweet potato as their first vegetable because it’s sweet. Let’s not give them that sweet tooth,’ kind of idea. They’re a group of women—and I say ‘women’ because they’re generally women at the market—who are making similar decisions. But if you were a mum who was in a different group, who were all vaccinating, formula feeding—and there’s no judgment [by me] aligned with this either—rice cereal giving at three months of age, etcetera, etcetera, then you would be supported by that similar group. But if you were the one mum in there doing the complete opposite, I reckon you’d be hightailing it out of that group and finding the group that aligns with you. Then we find these different groups of, generally, mums.”

Kavita suggests that the practices and dispositions and hence habitus (high forms of symbolic capital) are clear to all—criteria for membership of the social group, and markers of distinction from other social groups. Her narrative also emphasizes the homophily of the social field. When it comes to cultural practices that signify natural, health conscious parenting, everybody appears to be doing the same thing—that’s how you know whether you are doing the ‘right’ thing, and how you know whether you belong (or whether you should find a new social group instead). Hence parents gravitate towards each other as a form of bonding capital when they recognize a fellow member by their lifestyle markers. Cultural capital creates the centrifugal force for enhanced social capital and vice versa—social symbiosis. 

Some open questions that invite further research relate to the role of opinion leaders and charismatic individuals in setting and reinforcing the practices afforded high cultural capital within social groups. While our data present groups that individuals align with or—as Kavita describes—exit, it is more difficult to discern the role that particular individuals play in distinction making and enforcement according to symbolic capital. It is hard to pinpoint the role and power of individuals. Firstly, group dynamics rarely hinge on just one player. Secondly, vaccine rejecting parents place a high premium on critical thinking and personal decision-making, as we have documented with regard to this specific cohort [27] and others have documented elsewhere [46]. We think that this means parents are unlikely to disclose influence by or ‘followership’ of others, since this would imply that they were not thinking for themselves. Nevertheless, we envisage that parents who display proficiency in practices with high cultural capital may acquire the prestige and perceived expertise to opine and set agendas regarding other practices, such as vaccination. 

### 3.5 Implications for Interventions

One key implication of our research for intervention design emerges when we bring into the frame the theorization offered by philosopher and ethicist Mark Navin [30]. Navin suggests that all of us belong to epistemic communities, in which our general human tendencies to think and reason in particular ways (e.g. cognitive biases) play out in specific contexts, as shaped by those around us and the information-processing norms of those communities. He claims that as vaccine denialist epistemic communities form—such as the ones analyzed in this study—they develop and demonstrate particular epistemic vices. Most significantly, they function as supportive spaces, especially for people experiencing what we characterize as ‘habitus acceptance’ (unlike the adversarial culture of scientific epistemic communities). In this context, alternative forms of reasoning are not aired, nor existing forms challenged (unless those within are experiencing ‘disjunctive habitus’). These supportive communication practices do not challenge the ‘gut’ style instinctive responses to information that we all engage in, and which we usually then overlay with more sophisticated forms of reasoning that nevertheless prop up what we already ‘felt’. All this means that the reasoning processes of parents in vaccine rejecting epistemic communities are likely to be oriented towards maintaining beliefs that vaccines are dangerous or unnecessary. Our research thus invites consideration of how parents’ reasoning with regard to vaccines is informed by not only the *identity* of the social group and its associated capitals, but also its *practices* with regard to assessing and integrating information. Parents who want to fit in—or find that they already do—thus become part of the flawed epistemic practices of the group. On this basis, the maintenance of hegemonic anti-vaccine sentiments should not surprise us. 

What does this mean for interventions seeking to change how such parents feel about vaccines? Challenging how information flows through groups such as those analyzed in this article may involve intervening in the very dynamics of groups of individuals who are finding their way as parents, seeking to behave appropriately, and make friends. We should recognize that dissent within such a context—doing something different and particularly *saying* something different that makes the ‘doing’ public—would be troublesome when conformity to practices with high symbolic capital provides a passport to the group and the basis for forging friendships. There may be something to be gained by further understanding the dynamics of the ‘disjunctive habitus’ and how parents are made to feel within this context. Where distinctions are being drawn between them/us that make some parents feel between/betwixt social groups, they may be more open to vaccine-related information. This sub-group would likely be more receptive than those in more homophilic groups, experiencing ‘habitus acceptance’. However, we note that this may further tug the parents away from the peers who could most benefit (from a public health perspective) from engaging with the vaccination information they could potentially present. 

Resources providing parents with challenging but non-combative responses to vaccine-negative comments within their social networks could be one strategy for intervention designers to pursue. This would be a particularly useful resource given the current polarizing nature of discourse and dialogue between both sides of the vaccination debate in broader society. As noted above, we still need to learn more about the role of leaders, charismatic ‘experts’ and those who are afforded expert status by peers because they are proficient in other valued skills (baby-wearing or cooking organic food, for example). However, it is possible that encouraging vaccine supporting parents to share their skills in these domains could enhance their social and cultural capital to the extent that they are able to better challenge vaccine critical discourses in their friendship groups. 

### 3.6. Limitations

There are differences between the two communities in this study that in part derive from the sample techniques. As the Adelaide sample was obtained through an organic market and then through snowball sampling, we found already formed social networks. These people were purchasing organic food, so already possessed higher economic capital. Delving deeper, we found a shared set of highly valued symbolic capital surrounding natural living, schooling etc. On this basis, people at the heart of these social groups could expect to be accepted and receive positive reinforcement for their choices. This means they do not need to defend their practices as much—they are within social groups that have very similar practices and perspectives on what constitutes high and low symbolic capital. By contrast, the Fremantle study design excluded people who identified as anti-vaccination. Consequently, that sample consisted of the ring *around* the group of high symbolic capital parents who appeared, through the participants accounts, to define Fremantle ‘alternative’ parenting and the place of non-vaccination within it. We could see that central group of high symbolic capital parents, but in this context, we saw them through the eyes of those parents a step outside, experiencing habitus disjuncture and tug.

This research also has limitations common to all qualitative research. It is local, context specific and draws from a relatively small number of rich interviews rather than a large dataset. It may not be generalizable on these bases. A particular point to mention in this regard is that there may be differences between parents in developed world settings who reject vaccines based on perceptions and values regarding what is ‘natural’, and parents whose rejection instead relates to distrust of state and biomedical power and an inflated sense of personal responsibility. A recent study found that both purity and liberty matter to highly vaccine hesitant parents, and while US Republican political discourse emphasizes the latter, the political orientation did not affect the study’s results [47]. There is currently no published Australian data on the moral or political values of vaccine refusers that could inform the utility of such a purity/liberty distinction locally. However, we note that the majority of the Adelaide participants in this study, from whom such data was gathered, identified as voting for left or centre-left parties. 

## 4. Conclusions

Analyzing data from these two studies in Fremantle and Adelaide, we found a clear picture of how parents found themselves in vaccine hesitant and rejecting communities, how they learned from these communities, and how they experienced social norms around vaccination and other lifestyle attributes within them. Bourdieu’s concepts of symbolic capital, habitus and distinction, and Ingram’s taxonomy of destabilized habitus, habitus tug and disjunctive habitus, helped us to understand and elaborate the social processes, uncertainties and difficulties in the various vaccine-related journeys. Amongst the Fremantle participants, there were discussions of the social influences to refuse vaccines, and the emergence of a socially awkward space for those who were part of the community but accepted some or all vaccines. By contrast, the Adelaide participants, many of whom were more strident and homogenous in their rejection of vaccines, depicted like-minded communities with high bonding social capital within which their decisions were validated. Rather than the disjunctive habitus experienced by Fremantle participants, we conceptualize the Adelaide experiences as ‘habitus acceptance’. This was likely a product of the organic market and snowball recruitment technique, as many of the parents belonged to already formed social networks, including through the market. In both sites, however, parents presented an identity that included vaccine questioning and refusal as a ‘given’ marker of distinction. Considering the sociality of vaccine questioning and refusal reminds us that parents are encountering epistemic communities at the same time that they are making new friends and finding their path in a new phase of life. Interventions seeking to change or alter the flow of information through communal groups need to be designed with these matters in mind and may want to consider how parents can enhance and build upon their cultural capital in related areas of expertise in order to disseminate countering views that vaccinations are socially beneficial. We may all keep our silence at moments when we risk social opprobrium from those with whom we are seeking friendship and acceptance. Providing people with effective, evidence-based ways to respectfully disagree and introduce different perspectives would be an important start. 
